# Quantitative Genetic Analysis Reveals Potential to Genetically Improve Fruit Yield and Drought Resistance Simultaneously in Coriander

**DOI:** 10.3389/fpls.2017.00568

**Published:** 2017-04-20

**Authors:** Mostafa Khodadadi, Hamid Dehghani, Mokhtar Jalali Javaran

**Affiliations:** ^1^Plant Breeding Department, Faculty of Agriculture, Tarbiat Modares UniversityTehran, Iran; ^2^Biotechnology Department, Faculty of Agriculture, Tarbiat Modares UniversityTehran, Iran

**Keywords:** assimilate, coriander, drought escape, gene action, root, transpiration, water deficit stress

## Abstract

Enhancing water use efficiency of coriander (*Coriandrum sativum* L.) is a major focus for coriander breeding to cope with drought stress. The purpose of this study was; (a) to identify the predominant mechanism(s) of drought resistance in coriander and (b) to evaluate the genetic control mechanism(s) of traits associated with drought resistance and higher fruit yield. To reach this purpose, 15 half-diallel hybrids of coriander and their six parents were evaluated under well-watered and water deficit stressed (WDS) in both glasshouse lysimetric and field conditions. The parents were selected for their different response to water deficit stress following preliminary experiments. Results revealed that the genetic control mechanism of fruit yield is complex, variable and highly affected by environment. The mode of inheritance and nature of gene action for percent assimilate partitioned to fruits were similar to those for flowering time in both well-watered and WDS conditions. A significant negative genetic linkage was found between fruit yield and percent assimilate partitioned to root, percent assimilate partitioned to shoot, root number, root diameter, root dry mass, root volume, and early flowering. Thus, to improve fruit yield under water deficit stress, selection of low values of these traits could be used. In contrast, a significant positive genetic linkage between fruit yield and percent assimilate partitioned to fruits, leaf relative water content and chlorophyll content indicate selection for high values of these traits. These secondary or surrogate traits could be selected during early segregating generations. The early ripening parent (P_1_; TN-59-230) contained effective genes involved in preferred percent assimilate partitioning to fruit and drought stress resistance. In conclusion, genetic improvement of fruit yield and drought resistance could be simultaneously gained in coriander when breeding for drought resistance.

## Introduction

Abiotic stresses will remain a serious challenge to environmental and agricultural systems (Pereira, 2016). Drought is generally considered as the most common limitation to plant productivity. Drought resistance is a common term for a complex phenomenon which has restricted meaning without reference to a specific plant × environment combination (Serraj et al., 2004). Two main drought resistance mechanisms of crops include drought tolerance and drought avoidance. Avoidance of the plant water deficit may occur through three strategies including escape, avoidance by maximizing water acquisition through large root systems and avoidance by maintaining water in the cells through early closing of stomata/or having a waxy layer on leaves. Escape can occur if plants flower early and complete their life cycle before the drought stress fully develops (Bray, 2007). It has been stated that there is an interaction between drought escape and avoidance through maintenance of water if flowering time and water use efficiency are correlated (Franks, 2011). Also, modifying flowering time is an evolutionary strategy adopted by plants to maximize the chances of reproduction under various stresses such as drought conditions (Kazan and Lyons, 2016). Another approach to achieve drought avoidance is allocation of assimilates to deeper roots which is correlated with cooler canopies and improved yield under drought (Lopes and Reynolds, 2010).

Cell membrane stability (CMS) under water deficit conditions is another physiological criterion for screening drought tolerant genotypes (Rahman et al., 2008). This trait is measured based on electrolyte leakage from leaf segments. CMS has been largely used as an indicator of tolerance to different abiotic stresses and it has revealed a significant relationship between tolerance evaluated by CMS and yield of the crops under certain field conditions (Tripathy et al., 2000; Rahman et al., 2008). CMS and leaf relative water content are traits that have been linked to drought-tolerance (Teulat et al., 2003). Relative water content provides a measure of plant water-status and closely reflects the balance between water supply to the leaf and transpiration rate (Teulat et al., 2003).

Shoot traits associated with drought tolerance include rapid ground coverage and early growth vigor: leaf properties such as reduced leaf area, waxy leaf, leaf angles inclined more closely to stem and lower numbers of stomata. Likewise, root characteristics such as root system size, root morphology, root depth, root length, root density, root hydraulic conductance, as well as other functions, define and meet the transpiration demands of the crops (Sarker et al., 2005). Because of the technical difficulties of measuring root systems, they have been referred to as the “hidden half” of plants (Waisel et al., 2002). Recently, it has been highlighted that looking at roots in crops will be an important part of the second green revolution in agriculture (Gewin, 2010). Compared to shoot-related traits, integration of drought tolerance related root traits in breeding for drought tolerance has been comparatively less successful (Manschadi et al., 2008). As discussed by Farooq et al. (2009) drought tolerance is complex and several morphological, physiological and molecular mechanisms are usually involved. These mechanisms are often involved in determining transpiration efficiency under drought condition.

Transpiration efficiency is the genetic component of water-use efficiency (Vadez et al., 2014) which is determined as the total dry mass produced per unit of water transpired. Transpiration efficiency is an important crop feature, especially when water resources are limiting (Haefele et al., 2009). Genotypes that achieve high transpiration efficiency are likely to perform better under water-deficit conditions (Jyostna-Devi et al., 2009). Root dry weight has been shown to have an important contribution to transpiration efficiency under well-watered and especially under drought conditions (Puangbut et al., 2009). The inherent root traits of a plant influence its ability to extract soil water resources in rain fed areas. Response of root growth to certain levels of water stress during development can improve drought avoidance by increasing water uptake from deeper soil layers (Manavalan et al., 2010; Ober et al., 2014). Also, chlorophyll content has recently been used for indirect evaluation of transpiration efficiency because it has a significant relationship with transpiration efficiency (Nageswara Rao et al., 2001; Sheshshayee et al., 2006).

The major environmental factor affecting transpiration efficiency is atmospheric evaporative demand, and consequently plant transpiration increase with increasing atmospheric vapor pressure deficit. Plant photosynthesis is proportional to the transpiration rate multiplied by vapor pressure deficit divided by a transpiration efficiency coefficient (Fletcher et al., 2007).

Coriander is a diploid cross-pollinated crop and is a member of Apiaceae family (Diederichsen, 1996). Coriander has long been grown in the Mediterranean region, Asia Minor, southern Europe, and the Caucasus (López et al., 2008). Of the 60 crops producing essential oils, coriander has the most annual production volume (710 t) and value (49,700,000 US$) among 21 commercially established Apiaceae taxa essential oils producing crops in 2005 (Evergetis and Haroutounian, 2014). The major ingredients of dried fruits are fiber 23–36%, carbohydrates 13–20%, fatty oil 16–28%, proteins 11–17%, and essential oil 1–1.5% (Weiss, 2002; Msaada et al., 2009). Coriander is mainly cultivated for fruit production and is considered as a possible new oil seed crop (Sriti et al., 2010). In some studies, it was revealed that coriander production is significantly decreased by water deficit stress (Ghamarnia and Daichin, 2013; Hassan and Ali, 2014; Angeli et al., 2016).

Genetic loci that confirm productivity in stressed environments exist within the germplasm of plants and their wild relatives that are adapted to extreme environments (Mickelbart et al., 2015). Four regions of origin of variation were identified for coriander including the Near East, Ethiopia, Caucasia, and India (Diederichsen, 1996). Iran is located close to the regions of origin and it is expected that Iranian endemic coriander genotypes may contain genes involved in environmental stress tolerance.

The natural variation of crops is a genetic reservoir for abiotic stress adaptation (Pereira, 2016) and selection of donors containing genes involved in drought resistance is the first step to gain key knowledge about the genetics of drought resistance-related traits that is necessary to design successful breeding programs for improving them (Khodadadi et al., 2016a). Breeding for earliness may allow crops to escape terminal water deficit and access sufficient soil water during flowering and grain (fruit) filling stage (Nakhforoosh et al., 2015).

Variation in the general combining ability (GCA)/specific combining ability (SCA) ratio is useful in estimating whether existing variance is due to additive, non-additive or both types of gene action. When GCA/SCA ratio is substantially < 0.5 this indicates that the trait is predominantly controlled by non-additive gene action. Conversely, when GCA/SCA ratio is substantially higher than 0.5 this indicates that the trait is predominantly controlled by additive gene action. If the GCA/SCA ratio is ~0.5 then both additive and dominant gene action are controlling the trait (Baker, 1978). Heritability in the broad sense is determined as the proportion of phenotypic variation that is due to all genetic effects (additive and dominance effects; Holland et al., 2003). Heritability in the narrow sense is determined as the proportion of phenotypic variation among individuals in a population that is due to heritable genetic effects (main additive and additive × additive epistatic effects; Holland et al., 2003).

There is limited information about mechanism(s) of drought resistance and the genetics controlling drought resistance in coriander. The main objective of this research was to investigate the genetics of root attributes, transpiration efficiency and assimilate partitioning and any genetic linkage between these traits and drought escape in coriander.

## Materials and methods

### Plant material and growth conditions

A preliminary experiment to screen Iranian endemic coriander genotypes for drought resistance was carried out in 2013 at the agricultural faculty of Tarbiat Modares University, Iran and six genotypes were identified to be used as parents in the current study (Khodadadi et al., 2016a). Name, origin and characteristics of the six parents are presented in Table 1. Diallel crosses without reciprocals were made between the six parents in 2014. Parents and 15 hybrids were evaluated in 2015 as detailed below.

**Table 1:** **Genotype codes, accession numbers, origin, climate of origin, and response to drought stress**.

**Genotype**	**Parental code**	**Origin**	**Origin climate based on koppen climate classification**	**Relationship with genetic diversity origins considering leaf and fruit shape (Diederichsen, 1996)**	**Characteristics**
TN-59-230	P_1_	Bushehr	Hot desert (BWh)	Similar to Near Eastern type	Highly drought tolerant but low yielding
TN-59-160	P_2_	Mazandaran	Cold semi-arid (BSk)	Similar to Caucasian type	Drought tolerant and relatively high yielding
TN-59-353	P_3_	Markazi	Cold desert (BWk)	Similar to Ethiopian type	Drought tolerant and relatively high yielding
TN-59-80	P_4_	Isfahan	Cold desert (BWk)	Similar to Ethiopian type	Drought susceptible
Commercial	P_5_	Mahdasht	Cold semi-arid (BSk)	Similar to Indian type	Drought susceptible
TN-59-158	P_6_	Hamadan	Cold semi-arid (BSk)	Similar to Indian type	Highly drought susceptible

### Glasshouse experiment

#### Provision of the lysimeters and growth conditions

The plants were grown in lysimeters, consisting of PVC cylinders (20 cm diameter, 100 cm height) which contained a blend of sandy loam (3:2 v/v basis) and well ground compost (3:1 v/v basis). Two layers of plastic mesh (1 × 1 mm) were used as end plates allowing water drainage. The initial weights of filled cylinders were measured using a Mahak balance apparatus with 100 kg capacity and an accuracy of ±10 g. The cylinders were kept on mesh platforms. Buckets were then attached to the bottom of cylinders and the junction point between each bucket and cylinder was sealed using cellophane to prevent evaporation of drainage water from the buckets (Khodadadi et al., 2016b). Plant growth was carried out in a glasshouse with 14-h photoperiod, mean irradiance of 250 μmol m^−2^ s^−1^, 22–31°C mean temperature (T), 30–55% relative air humidity (RH). Glasshouse air T and RH were recorded three times a day, 07:00, 14:00, and 21:00 h. Temperature and relative humidity were measured using a Testo 625 humidity/temperature meter (Testo Inc., Sparta, NJ 07871, USA) in both glasshouse and field experiments. Air T and RH values were used to calculate atmospheric VPD (Fletcher et al., 2007) based on the formula suggested by Jones (2013). Glasshouse vapor pressure deficit status from 15 April 2015 to 8 July 2015 is presented in Supplementary Figure 1A.

#### Sowing and crop management

Seeds were thoroughly surface sterilized for 5 min in 10% sodium hypochlorite solution and then in 96% ethanol for 1 min and washed in sterile distilled water. Seeds were treated with fungicide to remove any seed borne diseases. Prior to planting, all the cylinders received 2 L of water to bring the soil profile up to field capacity. Subsequently, seeds were planted at a density of three per cylinder and later thinned to one plant per cylinder. To withhold evaporation from the soil surface (Ratnakumar and Vadez, 2011), the soil surface of the cylinders was mulched with aluminum foil. A split plot experiment based on a randomized complete block design with three replications was used in the glasshouse experiment. A set of genotypes (parents and their hybrids) were subjected to well-watered (WW) and water deficit stressed (WDS) treatments. WW plants were kept in soil maintained at field capacity moisture for the entire experiment while WDS plants were irrigated similarly to WW plants up until stem elongation, and from stem elongation to the flowering stage kept in 50% of field capacity until the start of flowering after which water was withheld. The amount of water (ml) used to irrigate of each cylinder was recorded. To improve soil fertility, the third, fourth, and fifth irrigations were done with 500 ml (2 g l^−1^) fertilizer solution (Greenline NPK-20-20-20, Germany). At the end of experiment, cylinders were immediately weighed after plants were harvested from atop the soil surface. Then the amount of transpired water (TW) was calculated for each genotype according to Equation (1). Results are shown in Supplementary Figure 2A.

(1) TW=TWU−(FWC−IWC)−DW

where TWU, FWC, IWC, and DW are total water (ml) used, final and initial weight (g) of cylinder and drainage water (ml).

### Field experiment

A yield trial (using the same 21 genotypes used in the glasshouse trial) was conducted consisting of two experiments in close proximity to each other at the research field of Tarbiat Modares University (51° 09′ E; 35° 44′ N; altitude 1,265 m), Iran during the period from the start of April to the end of June 2015. A randomized complete block design with three replications was used. Each plot consisted of three rows of five plants in each row. The spacing between rows was 45 cm and between plants 30 cm. In experiment 1, genotypes were kept WW during entire experiment. In experiment 2, watering was similar to experiment 1 until commencement of the flowering stage after which no further water was added (WDS).

Field soil properties are presented in Supplementary Table 1. Field atmospheric vapor pressure deficit, rainy days and the corresponding precipitation are presented in Supplementary Figure 1B. Air temperature and relative humidity ranged 20.1–26.0°C and 16.9–21.9%, respectively, at 07:00 h; 25.1–46.0°C and 3.0–20% at 14:00 h; 19.6–32.4°C and 14.2–24.8% at 21:00 h. Watering date and frequency are shown in Supplementary Figure 2B for each of genotype.

### Trait measurements

Traits including root dry mass (g), root length (cm), root number (No.), root diameter (mm), root volume (ml), fruit yield (g), transpiration efficiency (g ml^−1^), percent assimilate partitioned to root (%), percent assimilate partitioned to shoot (%), percent assimilate partitioned to fruit (%), chlorophyll content, leaf relative water content (%), CMS (%) and flowering time (d) were measured in the glasshouse experiment and also fruit yield was measured in the field experiment. All measurements were taken on single plants in each experimental unit (each cylinder) except for field data which were means of five samples in each plot. In both glasshouse and field experiments, each water regime contained three replications for each genotype. Shoots, and roots were dried to constant weight in a hot air oven at 70°C and weighed (Ratnakumar and Vadez, 2011).

For easily extracting roots from growth medium the cylinders were put in a water tank for 2 h and then the contents of cylinder (growth medium + roots) was slowly slid into the water and roots were simultaneously washed. The root extraction was done when plant leaves were beginning to dry but stems and roots remained relatively fresh and flexible. After root extraction, the main root branches were counted and expressed as root number.

As described by Vadez et al. (2011) transpiration efficiency was calculated as the ratio of the total biomass to the sum of transpired water for each cylinder during the experiment. Percent assimilate partitioned to root (PAPR), percent assimilate partitioned to shoot (PAPS) and percent assimilate partitioned to fruit (PAPF) were calculated according to Equations (2–4).

(2) $$\text{PAPR}=\frac{{\text{TE}}_{\text{R}}}{{\text{TE}}_{\text{TB}}}\times \text{100}$$

(3) $$\text{PAPS}=\frac{{\text{TE}}_{\text{S}}}{{\text{TE}}_{\text{TB}}}\times \text{100}$$

(4) $$\text{PAPF}=\frac{{\text{TE}}_{\text{F}}}{{\text{TE}}_{\text{TB}}}\times \text{100}$$

where TE_TB_, TE_R_, TE_S_, and TE_F_ are transpiration efficiency based on total biomass, root mass, shoot mass, and fruit mass, respectively. Chlorophyll content was estimated using the SPAD-502 Minolta Co., Ltd., Osaka, Japan in the fruit filling stage when severe drought stress appeared. Leaf relative water content was measured as described by Lafitte (2002), leaf sampling was done on all basal leaves, leaves of the middle of the stem and upper leaves of each plant and then placed in a pre-weighted plastic centrifuge vial. The vials were placed on ice, taken to a laboratory and weighted immediately. The vials were then filled with distilled water, recapped, and stored in dark 4°C room for 24 h. The next morning, leaves were blotted dry with paper towels for about 30 s per sample, and were weighted immediately to record fully turgid weight. Then samples were dried at 70°C to constant weight. Leaf sampling was done during the fruit filling stage when severe drought stress appeared in the morning after the dew had dried. Using the recorded weights, leaf relative water content (RWC) was calculated according to Equation (5) (Turner, 1986).

(5) $$\text{RWC}(\%)=\frac{\text{FW}-\text{DW}}{\text{TW}-\text{DW}}\times \text{100}$$

where FW, TW, and DW are sample fresh weight, turgid weight and dry weight, respectively. To measure CMS a method used by Rahman et al. (2008) was followed. Sampling was done on the same date as sampling for leaf relative water content. Samples were washed with deionized water to remove surface contamination and carefully blotted dry. Twenty 0.5 cm^2^ leaf discs were made from the bulked sample and submerged in 10 mL of deionized water in 20 mL screw-cap vials and kept at room temperature in the dark overnight. Then, conductance of the sample solutions was measured with a conductivity meter (Model, CR-30, Colorado, Denver instrument). Subsequently, the samples were autoclaved and total conductance of them was measured after vigorously shaking of vials to mix the contents. All measurements were recorded at 25°C. CMS was estimated as reciprocal of relative cell injury using the formula (Equation 6) suggested by Blum and Ebercon (1981).

(6) $$\text{CMS}(\%)=\frac{\text{1}-\frac{{\text{T}}_{\text{1}}}{{\text{T}}_{\text{2}}}}{\text{1}-\frac{{\text{C}}_{\text{1}}}{{\text{C}}_{\text{2}}}}\times \text{100}$$

where T_1_ and T_2_ are drought stressed sample conductance before autoclaving and drought stressed sample conductance after autoclaving, and C_1_ and C_2_ are well-watered sample conductance before autoclaving and well-watered sample conductance after autoclaving, respectively.

### Statistical analyses

Data validity and conformity to all hypothesis of analysis of variance were checked for all measured traits. A split-plot design with linear statistical model of y_ijk_ = μ + τ_i_ + β_j_ + τβ_ij_ + γ_k_ + βγ_jk_ + ε_ijk_ was used to analyze the glasshouse experiment data. Also, a combined analysis of variance linear model of y_ijk_ = μ + β_j_ + τβ_ij_ + γ_k_ + βγ_jk_ + ε_ijk_ was applied to analyze the field data. Where μ, τ_i_, β_j_, and γ_k_ are overall mean, block (replication) effect, water regime effect and genotype (6 parents plus 15 hybrids) effect, respectively. In the glasshouse experiment the τβ_ij_ and ε_ijk_ are main-plot error and sub-plot error, and in the field experiment these are main-experiment error and sub-experiment error, respectively. y_ijk_ represents the dependent variable and β_j_ and γ_k_ the independent variables.

GCA is the average efficiency of a genotype in hybrid combination and SCA measures whether certain combinations do comparatively better or worse than would be expected on the basis of the average efficiency of the genotypes involved (Griffing, 1956). The analysis of variance for GCA and SCA effects was carried out according to Griffing (1956) method 2, model 1 with statistical model of $x_{ij}=u+g_{i}+g_{j}+s_{ij}+\frac{1}{bc}\sum_{k}\sum_{l}e_{ijkl}$, where u, g_i_, g_j_, s_ij_, and e_ijkl_ are population mean, GCA effect of *i*th parent, GCA effect of *j*th parent, SCA effect of *i*th × *j*th hybrid and residual error of the *ijkl*th observation, respectively. Diallel analysis was done using the SAS program developed by Zhang et al. (2005). The $\sigma_{\text{g}}^{2}$ (GCA), $\sigma_{\text{s}}^{2}$ (SCA) and their variances were estimated for the random-effects model to calculate $\sigma_{\text{A}}^{2}$ (additive variance), $\sigma_{\text{D}}^{2}$ (dominance variance), and h^2^ (heritability; Zhang et al., 2005). GCA/SCA ratio was computed according to the method suggested by Baker (1978) (Equation 7).

(7) $$\text{GCA}/{\text{SCA}}_{\text{ratio}}=\frac{\text{2}\sigma_{\text{g}}^{2}}{\text{2}\sigma_{\text{g}}^{2}+\sigma_{\text{s}}^{2}}$$

Broad-sense heritability (${\text{h}}_{\text{B}}^{2}$) and narrow-sense heritability (${\text{h}}_{\text{N}}^{2}$) were calculated according to Equations (8, 9).

(8) $${\text{h}}_{\text{B}}^{2}=\frac{\sigma_{\text{A}}^{2}+\sigma_{\text{D}}^{2}}{\sigma_{\text{A}}^{2}+\sigma_{\text{D}}^{2}+\sigma_{\text{E}}^{2}}$$

(9) $${\text{h}}_{\text{N}}^{2}=\frac{\sigma_{\text{A}}^{2}}{\sigma_{\text{A}}^{2}+\sigma_{\text{D}}^{2}+\sigma_{\text{E}}^{2}}$$

where $\sigma_{\text{E}}^{2}$ is error variance. Genotypic correlation coefficients were calculated according to Holland (2006). SAS (2003) and Excel (2013) software were used for data analysis and making graphs, respectively.

## Results

### Root traits

**Figure 1:**
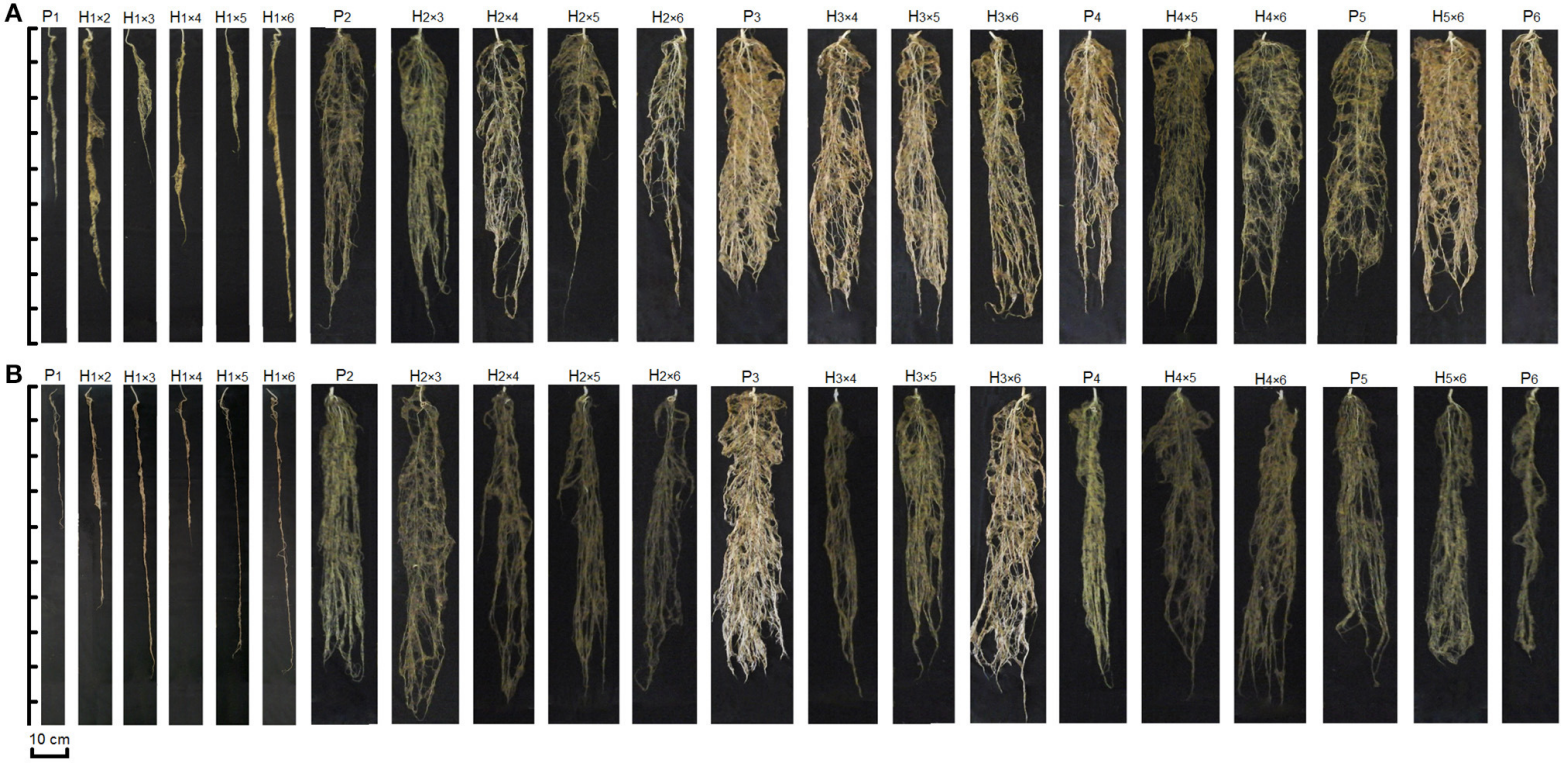
**Root shape of genotypes evaluated in lysimetric system. (A)** Root shape of genotypes under well-watered. **(B)** Root shape of genotypes under water deficit stressed. P_1_–P_6_: six parental coriander genotypes; H_1×2_–H_5×6_: 15 half-diallel hybrids.

Parental genotypes (P_1_, P_2_, P_3_, P_4_, P_5_, and P_6_) and hybrids exhibited high variation for root structure such as rooting depth and distribution (Figure 1). The analysis of variance for root traits indicated significant differences among genotypes (Table 2). Water treatment had a significant effect on root length, root diameter and root dry mass. The genotype × water treatment interaction was significant for root diameter, root dry mass, and root volume (Table 2). Significant GCA and SCA effects for all measured root traits were detected (*P* ≤ 0.01). The GCA × water treatment effect was significant for root number, root diameter and root dry mass, and SCA × water treatment was significant for root diameter, root dry mass and root volume (Table 2). Also, root length in WDS was significantly higher than WW condition (*P* ≤ 0.002).

**Table 2:** **Estimated variance for replication (rep), water treatment (WT), genotype (G), G × WT, general combining ability (GCA), specific combining ability (SCA), GCA × WT, SCA × WT, and error effects**.

**Sources of variation**	**Glasshouse experiment**														**Field experiment**	
	***df***	**RL**	**RN**	**RD**	**RDM**	**RV**	**TE**	**PAPR**	**PAPS**	**PAPF**	**FYG**	**CC**	**RWC**	**CMS**	***df***	**FYF**
Rep	2	775.5^**^	7.5^ns^	0.5^ns^	0.7^*^	8.9^ns^	1.0 E ^−7^	0.3^ns^	57.5^ns^	60.5^ns^	5.2^*^	32.0^ns^	9.8^ns^	42.6^ns^		
WT	1	11077.5^**^	2.4^ns^	25.8^**^	2.7^**^	1.6^ns^	1.3 E ^−5^**^^	975.5^**^	4178.7^**^	9192.3^**^	773.8^**^	6831.7^**^	31746.8^**^		1	632.5^**^
Rep (WT)	4	292.2	4.1	0.6	2.4	10.2	1.1 E ^−6^	5.0	108.3	110.9	6.2	80.4	2.2		4	6.6
Genotype (G)	20	1885.8^**^	31.5^**^	28.7^**^	3.81^**^	102.6^**^	1.0 E ^−6^**^^	34.5^**^	1746.0^**^	2129.1^**^	8.7^**^	118.5^**^	287.1^**^	902.3^**^	20	33.0^**^
G × WT	20	147.5^ns^	3.6^ns^	1.7^**^	0.6^**^	11.6^*^	2.4 E ^−7^^ns^	49.1^**^	98.1^**^	241.2^**^	8.6^**^	34.7^ns^	436.6^**^		20	17.9^**^
GCA	5	5937.7^**^	102.3^**^	102.7^**^	12.9^**^	337.9^**^	1.2 E ^−6^**^^	101.7^**^	6029.9^**^	7653.4^**^	26.9^**^	251.8^**^	869.6^**^	2277.5^**^	5	26.0^**^
SCA	15	600.1^**^	7.7^**^	3.9^**^	0.7^**^	24.1^**^	9.7 E ^−7^**^^	11.8^ns^	321.9^**^	290.7^**^	2.7^*^	72.6^**^	92.5^**^	443.9^**^	15	35.0^**^
GCA × WT	5	137.7^ns^	7.3^*^	1.9^*^	1.1^**^	12.9^ns^	2.3 E ^−7^^ns^	134.5^**^	275.5^**^	762.8^**^	27.3^**^	71.7^*^	1348.6^**^		5	43.3^**^
SCA × WT	15	159.5^ns^	2.3^ns^	1.6^*^	0.4^**^	11.1^*^	2.4 E ^−7^^ns^	20.4^**^	39.1^ns^	66.7^ns^	2.5^*^	23.5^ns^	132.2^**^		15	9.7^*^
Error	80	105.4	3.0	0.77	0.2	5.8	3.2 E ^−7^	8.1	37.7	39.0	1.25	25.1	11.5	37.3	80	4.9

RL, root length; RN, root number; RD, root diameter; RDM, root dry mass; RV, root volume; FYG, fruit yield in the glasshouse; TE, transpiration efficiency; PAPR, percent assimilate partitioned to root; PAPS, percent assimilate partitioned to shoot; PAPF, percent assimilate partitioned to fruit; CC, chlorophyll content; RWC, leaf relative water content; CMS, cell membrane stability; FYF, fruit yield in the field.

**, *, and ns *are significant at 1 and 5% level of probability and not significant*.

The largest negative significant GCA values were estimated for P_1_ for all root traits in both WW and WDS conditions (Table 3). In the case of SCA, the largest negative significant SCA values were estimated for H_1×5_ for root length, root number, root diameter and root volume and for H_1×3_ for root dry mass in WW (Table 4).

**Table 3:** **Estimated general combining ability of the parents**.

**Water treatment**	**Parent**	**Glasshouse experiment**													**Field experiment**
		**RL (cm)**	**RN (No.)**	**RD (mm)**	**RDM (g)**	**RV (ml)**	**TE (g ml^−1^)**	**PAPR (%)**	**PAPS (%)**	**PAPF (%)**	**FYG (g)**	**CC**	**RWC (%)**	**CMS(%)**	**FYF (g)**
WW	TN-59-230 (P_1_)	−21.9^**^A	−2.4^**^	−3.1^**^	−0.73^**^	−4.3^**^	−3.0E^−4^**^^	0.5^ns^	−17.5^**^	17.1^**^	−2.6^**^	0.3^ns^	−0.6^ns^		−2.4^**^
	TN-59-160 (P_2_)	6.2^**^	0.3^ns^	0.6^**^	−0.04^ns^	−0.8^ns^	1.2E^−4^^ns^	−0.9^*^	−1.9^ns^	2.8^*^	1.9^**^	2.5^**^	−4.6^**^		1.6^**^
	TN-59-353 (P_3_)	4.3^ns^	−0.2^ns^	0.3^ns^	0.24^**^	1.2^*^	0.5E^−4^^ns^	−0.1^ns^	4.1^**^	−3.9^**^	0.3^ns^	0.3^ns^	1.8^**^		1.7^**^
	TN-59-80 (P_4_)	2.9^ns^	0.9^**^	−0.1^ns^	0.14^ns^	1.2^*^	0.2E^−4^^ns^	−0.1^ns^	3.0^**^	−2.9^*^	0.5^ns^	−0.5^ns^	1.1^ns^		0.1^ns^
	Commercial (P_5_)	2.4^ns^	0.6^*^	1.0^**^	0.23^**^	2.1^**^	0.3E^−4^^ns^	0.7^ns^	6.0^**^	−6.7^**^	−0.4^ns^	−2.2^**^	4.1^**^		−1.1^*^
	TN-59-158 (P_6_)	9.1^**^	0.8^**^	1.2^**^	0.16^ns^	0.6^ns^	1.3E^−4^^ns^	0.1^ns^	6.3^**^	−6.4^**^	0.2^ns^	−0.4^ns^	−1.8^**^		0.1^ns^
	Intercept	68.0	8.1	5.5	1.3	7.7	1.8E^−3^	7.3	56.34	36.4	5.6	30.4	84.3		8.1
	SE_Gi_; SE_Gi-Gj_ ±	1.6; 2.5	0.2; 0.4	0.2; 0.3	0.01;0.01	0.5; 0.8	7E^−5^; 1E^−4^	0.4; 0.6	1.1;1.8	1.2; 1.8	0.3; 0.4	0.8; 1.3	0.6; 0.9		0.5; 0.8
WDS	P_1_	−21.5^**^	−3.2^**^	−2.7^**^	−1.3^**^	−5.3^**^	−3.1E^−4^*^^	−6.1^**^	−26.9^**^	33.1^**^	0.3^**^	1.0^ns^	16.6^**^	17.1^**^	0.9^**^
	P_2_	4.5^ns^	−0.5^ns^	0.3^ns^	0.04^ns^	−1.4^**^	0.3E^−4^^ns^	1.2^*^	1.3^ns^	−2.6^*^	0.4^**^	5.6^**^	4.2^**^	5.4^**^	0.6^*^
	P_3_	−2.5^ns^	0.2^ns^	.4^*^	0.3^**^	2.8^**^	1.9E^−4^^ns^	0.4^ns^	7.8^**^	−8.2^**^	−0.2^**^	−0.5^ns^	−2.4^**^	−4.9^**^	−0.6^*^
	P_4_	2.9^ns^	1.0^**^	0.1^ns^	0.1^ns^	0.2^ns^	−0.1E^−4^^ns^	0.9^ns^	4.0^**^	−4.9^**^	−8.3E^−3^^ns^	−0.2^ns^	−4.8^**^	−9.8^**^	−0.1^ns^
	P_5_	3.9^ns^	1.8^**^	1.3^**^	0.5^**^	2.4^**^	1.5E^−4^^ns^	1.1^ns^	7.3^**^	−8.4^**^	−0.2^**^	−1.3^ns^	−5.9^**^	−2.9^**^	−0.4^ns^
	P_6_	12.6^**^	0.5^ns^	0.6^**^	0.3^**^	1.3^**^	−0.5E^−4^^ns^	2.4^**^	6.5^**^	−8.9^**^	−0.3^**^	−4.6^**^	−7.7^**^	−4.8^**^	−0.4^ns^
	Intercept	86.6	8.4	6.6	1.6	7.5	2.5E^−3^	12.9	67.9	19.3	0.7	15.7	52.5	62.3	3.7
	SE_Gi_; SE_Gi-Gj_ ±	2.1; 3.3	0.4; 0.6	0.1; 0.2	0.1; 0.1	0.4; 0.6	1E^−4^; 2E^−4^	0.6; 1.0	1.2; 1.8	1.2; 1.8	0.1; 0.1	1.0; 1.6	0.7; 1.0	1.1, 1.8	0.3; 0.4

*RL, root length; RN, root number; RD, root diameter; RDM, root dry mass; RV, root volume; FYG, fruit yield in the glasshouse; TE, transpiration efficiency; PAPR, percent assimilate partitioned to root; PAPS, percent assimilate partitioned to shoot; PAPF, percent assimilate partitioned to fruit; CC, chlorophyll content; RWC, leaf relative water content; CMS, cell membrane stability; FYF, fruit yield in the field; WW, well-watered; WDS, water deficit stressed*.

A showing significance level relative to intercept;

**, *, and ns *are significant at 1 and 5% level of probability and not significant*.

*SE_Gi_ ±: standard error for any GCA effect; SE_Gi-Gj_ ±: standard error of the difference between any two GCA effects*.

**Table 4:** **Estimated specific combining ability of the F_1_ generation**.

**Water treatment**	**Hybrid**	**Glasshouse experiment**													**Field experiment**
		**RL (cm)**	**RN (No.)**	**RD (mm)**	**RDM (g)**	**RV (ml)**	**TE (g ml^−1^)**	**PAPR (%)**	**PAPS (%)**	**PAPF (%)**	**FYG (g)**	**CC**	**RWC (%)**	**CMS (%)**	**FYF (g)**
WW	H_1×2_	4.8^ns^^A^	2.7^**^	0.2^ns^	0.3^ns^	5.1^**^	0.4E^−4^^ns^	1.7^ns^	0.6^ns^	−2.2^ns^	0.1^ns^	4.1^ns^	4.9^**^		−0.7^ns^
	H_1×3_	−11.0^*^	−0.8^ns^	−1.0^*^	−0.6^*^	−3.5^*^	−6.9E^−4^**^^	−1.7^ns^	−9.4^**^	11.1^**^	−1.0^ns^	2.8^ns^	0.3^ns^		1.3^ns^
	H_1×4_	−0.5^ns^	−0.9^ns^	−0.3^ns^	−0.2^ns^	−1.6^ns^	−4.8E^−4^*^^	1.2^ns^	−5.0^ns^	3.8^ns^	−0.5^ns^	3.1^ns^	−5.4^**^		1.1^ns^
	H_1×5_	−13.3^**^	−1.9^**^	−1.9^**^	−0.4^*^	−3.6^*^	8.4E^−4^**^^	2.1^ns^	−8.5^**^	6.4^ns^	−1.2^ns^	3.1^ns^	1.2^ns^		−0.6^ns^
	H_1×6_	20.9^**^	−0.5	−0.4^ns^	0.4^ns^	1.0^ns^	6.3E^−4^**^^	3.9^**^	−7.2^*^	3.3^ns^	1.7^ns^	4.5^*^	−3.5^*^		2.3^ns^
	H_2×3_	11.8^**^	−0.5^ns^	−0.8^ns^	0.1^ns^	−1.1^ns^	−0.3E^−4^^ns^	0.8^ns^	−4.1^ns^	3.3^ns^	1.1^ns^	3.6^ns^	11.0^**^		6.2^**^
	H_2×4_	−2.6^ns^	−1.3^ns^	−1.2^*^	−0.5^*^	−2.9^*^	−1.3E^−4^^ns^	−1.4^ns^	−11.1^**^	12.4^**^	1.5^ns^	2.3^ns^	−1.3^ns^		1.1^ns^
	H_2×5_	0.6^ns^	1.4^ns^	−0.8^ns^	−0.3^ns^	−2.4^ns^	4.7E^−4^*^^	−2.4^*^	5.5^ns^	−3.1^ns^	0.5^ns^	−2.6^ns^	2.3^ns^		2.4^ns^
	H_2×6_	−4.7^ns^	−0.9^ns^	−0.03^ns^	−0.1^ns^	−0.5^ns^	−1.7E^−4^^ns^	−0.8^ns^	−0.3^ns^	1.1^ns^	−0.03^ns^	−2.3^ns^	−5.2^**^		1.6^ns^
	H_3×4_	1.9^ns^	−0.8^ns^	−0.3^ns^	−0.1^ns^	−0.8^ns^	2.3E^−4^^ns^	−0.7^ns^	−2.8^ns^	3.4^ns^	1.8^*^	−2.4^ns^	4.7^**^		1.0^ns^
	H_3×5_	7.1^ns^	0.8^ns^	0.7^ns^	0.3^ns^	1.2^ns^	2.2E^−4^^ns^	0.02^ns^	5.3^ns^	−5.3^ns^	−0.3^ns^	−2.0^ns^	−4.5^**^		−3.5^*^
	H_3×6_	4.1^ns^	0.2^ns^	−1.4^**^	−0.2^ns^	−0.7^ns^	−0.3E^−4^^ns^	−1.2^ns^	−4.7^ns^	6.0^ns^	1.5^ns^	−0.2^ns^	−1.8^ns^		0.1^ns^
	H_4×5_	3.7^ns^	−0.9^ns^	−0.03^ns^	0.4^ns^	2.9^*^	4.2E^−4^*^^	−0.1^ns^	7.1^*^	−7.0^*^	−0.4^ns^	2.4^ns^	−1.4^ns^		5.4^**^
	H_4×6_	1.7^ns^	0.8^ns^	0.9^ns^	0.2^ns^	0.8^ns^	−0.4E^−4^^ns^	0.9^ns^	2.2^ns^	−3.1^ns^	−0.7^ns^	1.1^ns^	−1.0^ns^		−1.5^ns^
	H_5×6_	−5.8^ns^	1.2^ns^	1.0^*^	−0.02^ns^	1.8^ns^	0.1E^−4^^ns^	−1.1^ns^	0.5^ns^	0.5^ns^	0.3^ns^	0.9^ns^	2.5^ns^		1.5^ns^
	SE_Si_; SE_Si–Sj_ ±	4.5; 5.1	0.6; 0.7	0.5; 0.6	0.04; 0.06	1.4; 1.6	2E^−4^; 2E^−4^	1.1; 1.3	3.1; 3.5	3.2; 3.6	0.8; 0.9	2.2; 2.5	1.6; 1.8		1.4; 1.6
WDS	H_1×2_	16.3^*^	0.6^ns^	0.4^ns^	−0.2^ns^	1.1^ns^	−2.5E^−4^^ns^	−2.7^ns^	−0.5^ns^	3.2^ns^	0.4^ns^	−4.1^ns^	3.4^ns^	−12.4^**^	0.5^ns^
	H_1×3_	−3.4^ns^	0.2^ns^	−1.1^**^	−0.4^ns^	−2.2^*^	0.5E^−4^^ns^	0.6^ns^	−9.7^**^	9.1^**^	0.3^ns^	4.8^ns^	15.0^**^	7.0^*^	2.6^**^
	H_1×4_	−0.7^ns^	−1.2^ns^	−0.9^*^	−0.3^ns^	−1.1^ns^	−8.4E^−4^*^^	−0.8^ns^	−10.9^**^	11.7^**^	0.04^ns^	−2.2^ns^	0.4^ns^	7.8^*^	2.0^*^
	H_1×5_	−6.1^ns^	−2.4^*^	−1.1^**^	−0.7^**^	−3.0^**^	−6.9E^−4^^ns^	−3.9^*^	−6.4^*^	10.3^**^	0.5^*^	6.4^*^	−3.7^*^	4.1^ns^	1.1^ns^
	H_1×6_	36.9^**^	−0.4^ns^	−0.6^ns^	−0.4^ns^	−1.2^ns^	3.6E^−6^^ns^	3.0^ns^	−10.8^**^	7.8^*^	0.4^ns^	2.7^ns^	3.8^*^	10.3^**^	2.2^**^
	H_2×3_	2.0^ns^	1.2^ns^	0.2^ns^	−0.01^ns^	−1.6^ns^	−0.6E^−4^^ns^	0.4^ns^	−1.8^ns^	1.4^ns^	0.2^ns^	4.7^ns^	−10.3^**^	−0.5^ns^	0.6^ns^
	H_2×4_	−5.0^ns^	−0.9^ns^	−0.5^ns^	−0.2^ns^	−0.5^ns^	−5.3E^−4^^ns^	1.5^ns^	−5.3^ns^	3.9^ns^	0.3^ns^	4.1^ns^	1.0^ns^	−19.8^**^	0.3^ns^
	H_2×5_	−5.1^ns^	0.3^ns^	0.3^ns^	−0.1^ns^	−0.2^ns^	2.6E^−4^^ns^	−0.9^ns^	3.9^ns^	−3.0^ns^	−0.2^ns^	1.1^ns^	5.7^**^	0.9^ns^	2.1^**^
	H_2×6_	−6.4^ns^	−0.4^ns^	0.4^ns^	0.5^ns^	0.2^ns^	0.6E^−4^^ns^	4.1^*^	0.3^ns^	−4.4^ns^	−0.4^ns^	−6.7^*^	−2.7^ns^	12.5^**^	−0.6^ns^
	H_3×4_	5.6^ns^	−0.7^ns^	−0.3^ns^	−0.1^ns^	−2.5^*^	3.6E^−4^^ns^	0.4^ns^	2.1^ns^	2.5^ns^	−0.01^ns^	−1.8^ns^	1.5^ns^	3.6^ns^	−0.4^ns^
	H_3×5_	5.2^ns^	−0.5^ns^	0.3^ns^	0.4^ns^	0.9^ns^	1.0E^−4^^ns^	0.5^ns^	2.1^ns^	−2.6^ns^	−0.2^ns^	−3.6^ns^	2.2^ns^	13.5^**^	−1.5^ns^
	H_3×6_	−12.1^ns^	1.5^ns^	0.7^ns^	0.4^ns^	3.7^**^	−4.2E^−4^^ns^	1.1^ns^	0.2^ns^	−1.3^ns^	−0.3^ns^	−0.7^ns^	4.0^*^	−7.7^*^	1.3^ns^
	H_4×5_	8.2^ns^	0.7^ns^	0.03^ns^	0.3^ns^	−2.8^**^	1.6E^−4^^ns^	3.1^ns^	−4.4^ns^	1.3^ns^	0.5^*^	2.5^ns^	9.1^**^	−16.5^**^	1.6^*^
	H_4×6_	−6.1^ns^	−1.0^ns^	0.2^ns^	−0.7^**^	0.9^ns^	−1.7E^−4^^ns^	−5.3^**^	0.3^ns^	2.0^ns^	0.3^ns^	−0.9^ns^	−5.5^**^	4.2^ns^	−0.2^ns^
	H_5×6_	0.1^ns^	1.9^ns^	−0.3^ns^	0.3^ns^	0.1^ns^	0.2E^−4^^ns^	0.9^ns^	0.3^ns^	−1.3^ns^	−0.1^ns^	4.3^ns^	2.8^ns^	11.4^**^	−0.1^ns^
	SE_Si_; SE_Si-Sj_ ±	5.9; 6.7	1.1; 1.2	0.4; 0.4	0.2; 0.2	1.0; 1.1	3E^−4^; 4E^−4^	1.7; 1.9	3.1; 3.6	3.2; 3.6	0.2; 0.3	2.9; 3.2	1.8; 2.1	3.1; 3.5	0.7; 0.8

*RL, root length; RN, root number; RD, root diameter; RDM; root dry mass; RV, root volume; FYG, fruit yield in the glasshouse; TE, transpiration efficiency; PAPR, percent assimilate partitioned to root; PAPS, percent assimilate partitioned to shoot; PAPF, percent assimilate partitioned to fruit; CC, chlorophyll content; RWC, leaf relative water content; CMS, cell membrane stability; FYF, fruit yield in the field; WW, well-watered; WDS, water deficit stressed*.

A showing significance level relative to intercept;

**, *, and ns *are significant at 1 and 5% level of probability and not significant. SE_Si_ ±: standard error for any SCA effect; SE_Si-Sj_ ±: standard error of the difference between any two SCA effects*.

The largest negative SCA was exhibited by H_3×6_ for root length and H_1×5_ for root number, root diameter, root dry mass and root volume in WDS (Table 4). Heritability estimates for root traits are presented in Table 5. Root traits had high GCA/SCA ratio ranged from 0.83 for root volume and root number to 0.91 for root diameter in WW and 0.78 for root length to 0.98 for root number in WDS conditions. Narrow-sense heritability of root traits ranged from 0.59 to 0.79 in WW and 0.57 to 0.86 in WDS. Narrow-sense heritability of root traits in WDS were higher than those in WW except for root length.

**Table 5:** **Analysis of combining ability; heritability estimates under well-watered (WW) and water deficit stressed (WDS); Variance of general ($\sigma_{\text{g}}^{2}$) and specific ($\sigma_{\text{s}}^{2}$) combining ability, broad sense heritability (${\text{h}}_{\text{B}}^{2}$), narrow sense heritability (${\text{h}}_{\text{N}}^{2}$), and GCA/SCA ratio**.

**Water treatment**	**Estimate**		**Glasshouse experiment**														**Field experiment**
			**RL**	**RN**	**RD**	**RDM**	**RV**	**TE**	**PAPR**	**PAPS**	**PAPF**	**FYG**	**CC**	**RWC**	**FT**	**CMS**	**FYF**
WW	GCA		2859.1^**^	37.2^**^	58.9^**^	3.4^**^	130.9^**^	6.2E^−7^**^^	7.5^ns^	1987.6^**^	1963.0^**^	52.1^**^	55.9^*^	221.8^**^	2005.2^**^		59.8^**^
	SCA		241.6^**^	5.1^**^	3.8^**^	0.4^*^	19.2^**^	5.8E^−7^**^^	11.4^*^	178.8^**^	160.7^**^	4.7^*^	42.3^*^	72.2^**^	59.51^**^		33.3^**^
	Error		77.1	1.6	1.0	0.2	7.6	1.4E^−7^	5.1	37.6	38.4	2.3	19.0	10.0	19.93		7.9
	$\sigma_{\text{g}}^{2}$	value	109.1^**^	1.3^**^	2.3^**^	0.12^**^	4.7^**^	2.0E^−9^^ns^	0.0^ns^	75.4^**^	75.1^**^	2.0^**^	0.6^ns^	6.2^*^	81.06^**^		1.1^ns^
		SE	30.85	0.37	0.65	0.03	1.33	5.6E^−10^	0.00	21.33	21.24	0.57	0.17	1.75	22.93		0.31
	$\sigma_{\text{s}}^{2}$	value	54.8^**^	1.1^**^	0.9^**^	0.06^*^	3.9^**^	1.5E^−7^**^^	2.1^*^	47.1^**^	40.8^**^	0.8^*^	7.8^*^	20.7^**^	13.2^**^		8.5^**^
		SE	5.17	0.10	0.08	0.01	0.37	1.4E^−8^	0.20	4.44	3.85	0.08	0.74	1.95	1.24		0.8
	${\text{h}}_{\text{B}}^{2}$	value	0.91	0.87	0.94	0.82	0.84	0.77	0.55	0.94	0.94	0.86	0.59	0.91	0.96		0.80
		SE	0.33	0.34	0.33	0.35	0.35	0.51	0.72	0.33	0.33	0.35	0.58	0.36	0.35		0.44
	${\text{h}}_{\text{N}}^{2}$	value	0.73	0.61	0.79	0.65	0.59	0.02	0.00	0.72	0.74	0.72	0.08	0.34	0.89		0.17
		SE	0.31	0.28	0.32	0.31	0.29	0.13	0.13	0.30	0.31	0.32	0.17	0.20	0.34		0.17
	GCA/SCA		0.89	0.83	0.91	0.89	0.83	0.05	0.00	0.86	0.88	0.91	0.24	0.55	0.96		0.34
WDS	GCA		3216.4^**^	72.4^**^	45.8^**^	10.7^**^	219.9^**^	7.6E^−7^^ns^	228.6^**^	4317.8^**^	6453.2^**^	2.2^**^	267.6^**^	1996.4^**^	2304.6^**^	2277.5^**^	9.5^**^
	SCA		518.1^**^	5.0^ns^	1.7^**^	0.7^**^	16.0^**^	6.2E^−7^^ns^	20.7^ns^	182.2^**^	196.7^**^	0.5^**^	53.8^ns^	152.6^**^	31.07^ns^	443.9^**^	11.5^**^
	Error		133.6	4.3	0.5	0.1	4.0	4.9E^−7^	11.1	37.9	39.7	0.2	31.1	12.9	18.31	37.3	2.1
	$\sigma_{\text{g}}^{2}$	value	112.4^**^	2.8^**^	1.8^**^	0.4^**^	8.5^**^	5.7E^−9^^ns^	8.7^**^	172.3^**^	260.7^**^	0.1^**^	8.9^**^	76.8^**^	94.70^**^	76.4^**^	0.7^ns^
		SE	31.79	0.79	0.51	0.11	2.40	1.6E^−9^	2.46	48.73	73.74	0.03	2.52	21.72	26.79	21.61	0.20
	$\sigma_{\text{s}}^{2}$	value	128.2^**^	0.2^ns^	0.4^**^	0.2^**^	4.0^**^	4.4E^−8^^ns^	3.2^ns^	48.1^**^	52.3^**^	0.1^**^	7.5^ns^	46.5^**^	4.25^ns^	135.5^**^	3.1^**^
		SE	12.09	0.02	0.04	0.02	0.38	4.1E^−9^	0.30	4.53	4.93	0.01	0.71	4.38	0.40	12.78	0.29
	${\text{h}}_{\text{B}}^{2}$	value	0.89	0.80	0.96	0.97	0.94	0.25	0.85	0.97	0.98	0.82	0.71	0.98	0.97	0.96	0.87
		SE	0.33	0.37	0.34	0.33	0.33	0.91	0.35	0.34	0.34	0.38	0.40	0.32	0.36	0.32	0.52
	${\text{h}}_{\text{N}}^{2}$	value	0.57	0.77	0.86	0.77	0.76	0.05	0.72	0.85	0.89	0.55	0.50	0.75	0.95	0.51	0.27
		SE	0.27	0.35	0.34	0.31	0.31	0.23	0.32	0.33	0.34	0.26	0.28	0.30	0.36	0.24	0.11
	GCA/SCA		0.78	0.98	0.95	0.89	0.89	0.34	0.92	0.93	0.95	0.80	0.83	0.87	0.99	0.69	0.47

RL, root length; RN, root number; RD, root diameter; RDM, root dry mass; RV, root volume; FYG, fruit yield in the glasshouse; TE, transpiration efficiency; PAPR, percent assimilate partitioned to root; PAPS, percent assimilate partitioned to shoot; PAPF, percent assimilate partitioned to fruit; CC, chlorophyll content; RWC, leaf relative water content; CMS, cell membrane stability; FT, flowering time; FYF, fruit yield in the field.

**, *, and ns *are significant at 1 and 5% level of probability and not significant. SE: Standard error*.

### Transpiration efficiency and assimilate partitioning traits

As expected, the water treatment had a significant effect on transpiration efficiency, percent assimilate partitioned to root, percent assimilate partitioned to shoot and percent assimilate partitioned to fruit traits. The ANOVA showed a significant genotypic effect for these traits (Table 2), and also a significant genotype × water treatment interaction for these traits with the exception of transpiration efficiency. These traits exhibited significant GCA and SCA effects except percent assimilate partitioned to root which had a significant GCA effect only. Assimilate partitioning traits showed a significant GCA × water treatment effect. SCA × water treatment effect was significant only for percent assimilate partitioned to root (Table 2).

**Figure 2:**
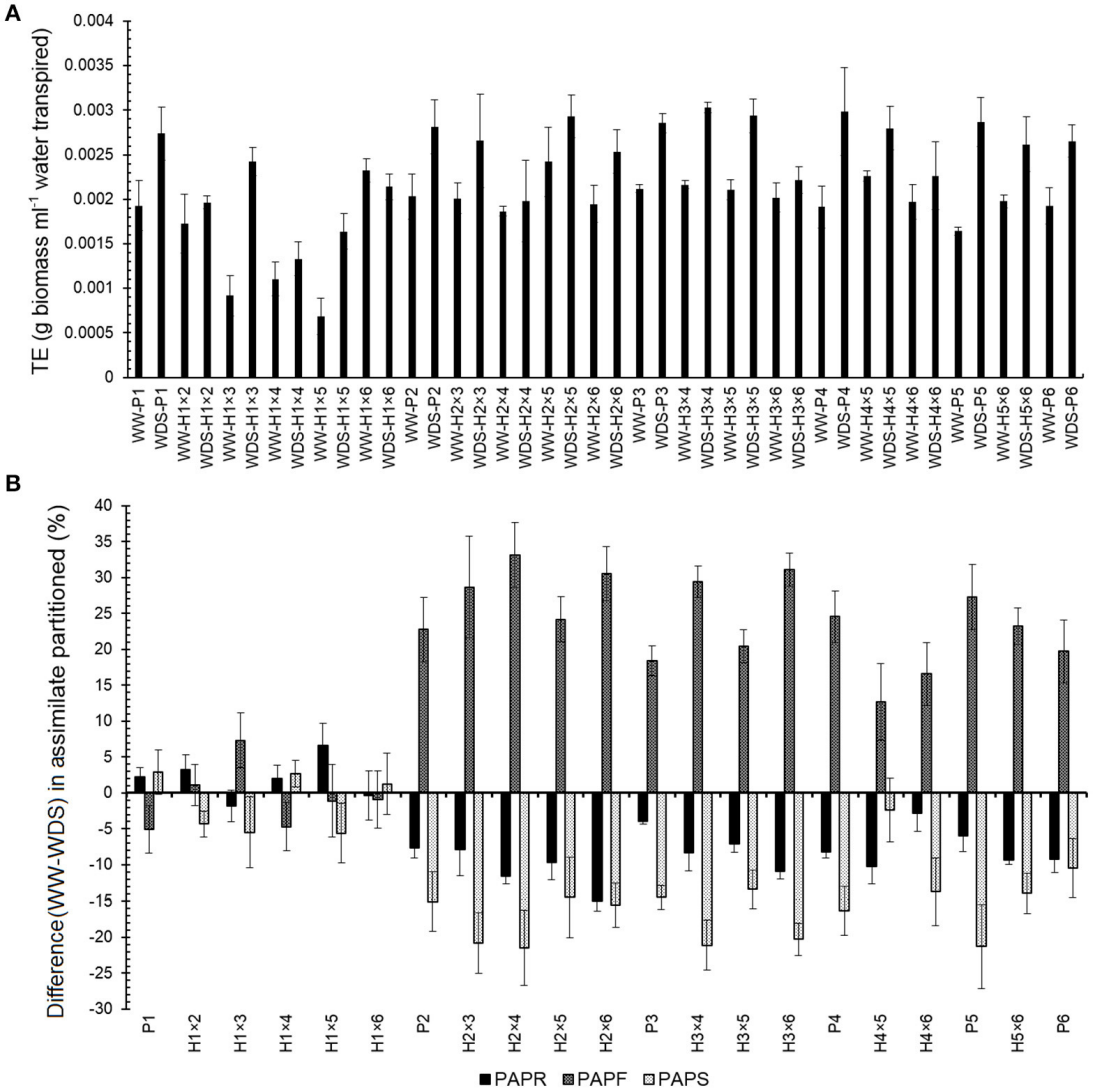
**Transpiration efficiency (g biomass ml^−1^ water transpired) and assimilate partitioning of coriander genotypes. (A)** Transpiration efficiency of coriander genotypes under well-watered (WW) and water deficit stressed (WDS). **(B)** Difference (WW-WDS) in assimilate partitioning of genotypes under well-watered (WW) and water deficit stressed (WDS). PAPR, PAPS, and PAPF are percent assimilate partitioned into root, shoot and fruit, respectively. P_1_–P_6_: six parental coriander genotypes; H_1×2_–H_5×6_: 15 half-diallel hybrids.

Transpiration efficiency in WDS was significantly higher than WW condition as shown in Figure 2A (students *t*-test, *t* = −4.5; *P* ≤ 0.001). Parental genotypes and hybrids exhibited different patterns of assimilate partitioning to root, shoot, and fruit in both WW and WDS conditions. P_1_ and related hybrids (H_1×2_, H_1×3_, H_1×4_, H_1×5_, and H_1×6_) had the highest assimilate partitioning to fruit and the lowest to root in both WW and WDS conditions while in other cases the highest assimilate partitioned was to the shoot except for H_2×4_ in WW condition (Figure 2B). The P_1_, H_1×4_, H_1×5_, and H_1×6_ showed more assimilate partitioning to fruit in WDS than those in WW (Figure 2B).

The GCA estimates showed that P_6_ (drought sensitive) had the highest value for transpiration efficiency and percent assimilate partitioned to shoot. In contrast, P_1_ (drought resistant) had the highest value for percent assimilate partitioned to fruit in WW condition. In WDS conditions, P_1_, P_6_, and P_3_ had the highest GCA for percent assimilate partitioned to fruit, percent assimilate partitioned to root and percent assimilate partitioned to shoot, respectively. In contrast, P_1_ had the largest negative GCA for percent assimilate partitioned to root and percent assimilate partitioned to shoot and P_6_ for percent assimilate partitioned to fruit (Table 3).

Results of SCA analysis for these traits indicated that H_1×5_, H_1×6_, H_2×5_, and H_2×4_ had the greatest positive and H_1×3_, H_2×5_, H_2×4_, and H_4×5_ had the greatest negative values for transpiration efficiency, percent assimilate partitioned to root, percent assimilate partitioned to shoot and percent assimilate partitioned to fruit in WW condition, respectively (Table 4). While in WDS, different results were observed, such that H_1×4_ showed the largest negative SCA for transpiration efficiency and percent assimilate partitioned to shoot but the highest SCA value for percent assimilate partitioned to fruit. H_2×6_ exhibited the largest negative value for percent assimilate partitioned to fruit and highest for percent assimilate partitioned to root. Maximum positive SCA of transpiration efficiency and percent assimilate partitioned to shoot calculated for H_3×4_ and H_2×5_, respectively, and the greatest negative SCA of percent assimilate partitioned to root calculated for H_4×6_ (Table 4). The heritability estimates of assimilate partitioning traits were higher in WDS than in WW condition (Table 5).

### Morphological, physiological, and phenological traits

According to ANOVA, water treatment, genotype, GCA, and SCA had significant effects on fruit yield in the glasshouse, chlorophyll content, leaf relative water content, CMS and fruit yield in the field (Table 2). GCA × water treatment was significant for all of these traits and also SCA × water treatment was significant for fruit yield in the glasshouse, leaf relative water content and fruit yield in the field (Table 2).

In WW condition, P_3_, P_2_, and P_5_ had the highest significant GCA for fruit yield in the field, fruit yield in the glasshouse and chlorophyll content, and leaf relative water content (Table 3). In WDS conditions, parents had different and in some case opposite GCA for these traits. P_1_ had the highest GCA for leaf relative water content, CMS and fruit yield in the field. P_2_, had the highest GCA for fruit yield in the glasshouse and chlorophyll content. P_6_ had the largest negative GCA for fruit yield in the glasshouse, chlorophyll content and leaf relative water content, P_4_ for CMS and P_3_ for fruit yield in the field.

In WW conditions, H_1×6_, H_1×2_, and H_2×3_ had the highest SCA for fruit yield in the glasshouse, chlorophyll content, and fruit yield in the field, respectively (Table 4). In WDS conditions, H_1×3_ showed significant positive SCA for leaf relative water content and fruit yield in the field (Table 4). H_1×5_ had a significant positive SCA for fruit yield in the glasshouse and chlorophyll content. The greatest negative SCA for leaf relative water content and fruit yield in the field were exhibited by H_2×3_ and H_3×5_, respectively.

GCA/SCA ratio for fruit yield in the glasshouse and leaf relative water content was higher than 0.55 and for chlorophyll content and fruit yield in field lower than 0.35 in WW condition. The GCA/SCA ratio for CMS was estimated at 0.69 (Table 5). Heritability estimates in WDS for these traits were similar to WW except for chlorophyll content and leaf relative water content which exhibited higher GCA/SCA ratio in WDS than in WW. The narrow-sense heritability (0.89 in WW; 0.95 in WDS) and GCA/SCA ratio (0.96 in WW; 0.99 in WDS) of flowering time was high in both water treatments indicating that this trait is governed by additive gene action.

### Genetic correlation among traits

Root traits had a significant negative correlation with percent assimilate partitioned to fruit in both WW and WDS conditions. Fruit yield in the glasshouse and fruit yield in the field showed a positive relation with root traits in WW while these relations were negative in WDS (Table 6). The relationship between transpiration efficiency and root traits was positive in both conditions. Contrasting direction of the relationships in WW and WDS conditions was indicated for percent assimilate partitioned to root, percent assimilate partitioned to fruit and transpiration efficiency, and also between leaf relative water content and fruit yield in the glasshouse (Table 6). Other contrasts in correlation were indicated between fruit yield in the glasshouse and fruit yield in the field were observed for percent assimilate partitioned to shoot, CMS, percent assimilate partitioned to fruit and transpiration efficiency in WW and WDS conditions (Table 6).

**Table 6:** **Genetic correlation coefficients and their standard error (SE) in parenthesis between percent assimilate partitioned to fruit (PAPF), transpiration efficiency (TE), fruit yield in the glasshouse (FYG) and fruit yield in the field (FYF) and other traits measured under well-watered and water deficit stressed**.

**Water treatment**	**Estimate**	**RL**	**RN**	**RD**	**RDM**	**RV**	**CC**	**RWC**	**CMS**	**PAPR**	**PAPS**	**PAPF**	**TE**	**FYG**
Well-watered	PAPF	**−0.80(0.10)**	**−0.87(0.07)**	**−1.0(0.03)**	**−0.95(0.03)**	**−0.98(0.04)**	**0.50(0.24)**	−0.21(0.23)	**0.70 (0.13)**	0.14(0.32)	**−0.99(0.01)**			
	TE	**0.76(0.13)**	**0.71(0.17)**	**0.66(0.17)**	**0.73(0.15)**	**0.66(0.18)**	−0.25(0.32)	−0.13(0.27)	−0.39(0.22)	−0.58(0.36)	**0.69(0.16)**	**−0.65(0.17)**		
	FYG	**0.87(0.07)**	**0.67(0.16)**	**0.61(0.17)**	**0.58(0.18)**	**0.45(.22)**	0.25(0.30)	−0.13(0.25)	−**0.47(0.19)**	−**0.6(0.29)**	**0.54(0.19)**	−**0.49(0.20)**	**0.65(0.17)**	
	FYF	**0.63(0.17)**	0.18(0.26)	0.25(0.24)	0.39(0.24)	0.19(0.26)	**0.69(0.23)**	0.06(0.26)	−0.34(0.23)	−0.38(0.33)	0.25(0.25)	−0.22(0.25)	**0.53(0.24)**	**0.80(0.13)**
	FT									−0.16(0.31)	**0.97(0.02)**	**−0.97(0.02)**		
Water deficit stressed	PAPF	**−0.68(0.14)**	**−0.95(0.05)**	**−0.98(0.02)**	**−0.98(0.01)**	**−0.90(0.05)**	0.38(0.24)	**0.88(0.05)**	**0.71(0.11)**	**−0.93(0.05)**	**−0.99(0.01)**			
	TE	0.32(0.44)	**0.98(0.46)**	**1.10(0.53)**	**1.17(0.54)**	**0.95(0.47)**	−0.51(0.57)	−0.79(0.49)	−0.47(0.43)	0.85(0.46)	**1.15(0.52)**	−**1.11(0.51)**		
	FYG	−0.19(0.26)	**−0.69(0.17)**	**−0.65(0.16)**	**−0.73(0.13)**	**−0.85(0.10)**	**1.11(0.15)**	**0.75(0.13)**	**0.44(0.22)**	−**0.49(0.21)**	**−0.69(0.14)**	**0.66(0.15)**	−**0.96(0.46)**	
	FYF	−0.02(0.26)	−**0.55(0.22)**	**−0.58(0.18)**	**−0.63(0.17)**	**−0.70(0.15)**	**0.68(0.20)**	**0.69(0.15)**	**0.42(0.21)**	−0.35(0.25)	**−0.67(0.15)**	**0.62(0.16)**	−1.49(0.78)	**0.93(0.10)**
	FT									**0.91(0.08)**	**0.97(0.01)**	**−0.98(0.01)**		

*RL, root length; RN, root number; RD, root diameter; RDM, root dry mass; RV, root volume; PAPR, percent assimilate partitioned to root; PAPS, percent assimilate partitioned to shoot; CC, chlorophyll content; RWC, leaf relative water content; FT, flowering time; CMS, cell membrane stability*.

*The bold underlined, bold and regular correlation coefficients are significant at 1, 5 % level of probability and not significant, respectively*.

The genetic relationship between flowering time and percent assimilate partitioned to root reversed from a negative correlation (*r* = −0.16) in WW to a positive correlation (*r* = 0.91) in WDS (Table 6). Furthermore, the relationships between days to flowering and percent assimilate partitioned to shoot and percent assimilate partitioned to fruit were positive and negative in both water regimes, respectively.

## Discussion

To mitigate the impact of water deficit stress, plants use different strategies such as anatomical, morphological, and physiological mechanisms to decrease transpiration, increase water absorption and reduce oxidative damage (Sayar et al., 2007). Root traits are involved in drought avoidance by increasing water uptake capacity, and flowering time is involved in drought escape. Assimilate partitioning is involved in the trade-off between drought escape and drought avoidance. Chlorophyll content, CMS and leaf relative water content are involved in drought tolerance. Therefore, to identify the major drought resistance mechanism(s) in coriander, genetics of inheritance of these traits and morphological productivity traits, as well as the genetic correlation between the traits were evaluated. If there is a significant genetic linkage between the drought stress resistance traits and percent assimilate partitioned to fruit, this could suggest that genetic improvement of fruit yield and drought resistance could be achieved simultaneously in coriander particularly if the genetic inheritance proved similar.

### Root traits

Roots are a hidden half of plants which play an important role in adaptation to drought stress. Large differences in root shape and attributes between parents and hybrids indicated that parent selection had produced the desired wide range in root traits. Root attributes were influenced by WDS, except for root number, indicating that water deficit in the rhizosphere led to genotypic responses in root growth pattern. Sayar et al. (2007) reported that root length and density are common features of drought stress resistant plants. Also Liu and Li (2005) concluded that reductions in root respiration and root biomass under severe water stress can improve drought resistance in wheat, results that are in accordance with the present study.

The significant GCA and SCA effects for all root traits indicate that both additive and dominance gene actions are involved in controlling these traits. A significant GCA × water treatment interaction effect suggests that additive gene actions for root number, root diameter and root dry mass can be altered in different water regimes. Also a significant GCA × water treatment interaction effect indicates that dominance gene actions for root diameter, root dry mass and root volume can be altered in different water regimes.

High values of GCA/SCA ratios for root traits suggest that the additive gene action is predominant in controlling these traits in both WW and WDS conditions. Therefore, selection for the improvement of these characters will be effective in early generations (Lal et al., 2006). Higher narrow-sense heritability of root number, root diameter, root dry mass, and root volume in WDS indicates that additive gene action had a more important role in WDS than WW in controlling root traits. Gowda et al. (2011) reported narrow-sense heritability estimates >50% for root diameter, root length, root dry weight and that these are polygenic traits in rice. These results for rice are in accordance with our results for coriander. Also, Sayar et al. (2007) estimated high narrow-sense heritability (0.81) for root length in durum wheat.

### Transpiration efficiency and assimilate partitioning traits

Lysimetric systems allowed precise evaluation of transpiration ability based on total biomass in our study. Transpiration efficiency is a genetic component of water use efficiency and depends on both genetic and environmental factors (Vadez et al., 2014). Result of *t*-test revealed that transpiration efficiency of coriander in this study was significantly increased under WDS, which is in contrast with previous results for *Arachis hypogaea* L. (Ratnakumar and Vadez, 2011). Lal et al. (2006) reported that partitioning of assimilate, as measured by harvest index, had the greatest effect on pod yield in *Arachis hypogaea* L. for water limited conditions. Therefore, a good knowledge of genetic systems controlling the expression of transpiration efficiency and assimilate partitioning is essential when devising breeding strategies for specific crops.

Our finding of significant GCA and SCA effects on transpiration efficiency, combined with the non-significant GCA × water treatment and SCA × water treatment, indicate that both additive and dominance gene actions are involved in expression of transpiration efficiency. In addition, the inheritance of transpiration efficiency was not significantly altered by different water regimes. Very low GCA/SCA ratio and high broad sense heritability indicate that dominance effects are important in controlling transpiration efficiency in WW.

Appropriate assimilate partitioning in water-limited environments is the key of stress resistance. Guan et al. (2010) reported that efficient carbon partitioning and early flowering time contributed to drought resistance and high harvest index. Therefore, to gain further insight into the nature of gene action in regulation of assimilate partitioning and flowering time under WW and WDS conditions, percentage assimilate partitioned into root, shoot and fruit were evaluated in coriander and compared to flowering time.

Our results indicate that both additive and dominant gene effects are involved in controlling percent assimilate partitioned to shoot and percent assimilate partitioned to fruit. Further, the additive effect of genes may be altered under different water treatments. According to heritability estimates presented in Table 5, percent assimilate partitioned to root in WW is controlled by dominant gene action. However, in WDS this trait is controlled by additive gene action. This indicates that regulation of assimilate partitioning into roots in different watering conditions were controlled by different genetic mechanisms. The high narrow-sense heritability and high GCA/SCA ratio for percent assimilate partitioned to shoot and percent assimilate partitioned to fruit have revealed mostly additive gene action involved in regulation of these traits under both irrigation regimes.

Despite low heritability of transpiration efficiency, heritability of percent assimilate partitioned to root, percent assimilate partitioned to shoot and percent assimilate partitioned to fruit were very high. This suggests that genetic regulation of partitioning of harvested carbon is more effective than genetic regulation of total carbon harvesting to differentiate drought resistant genotypes from drought sensitive genotypes under WDS conditions. Genetic regulation of partitioning of harvested carbon is also useful to identify drought resistance mechanism(s) in coriander. As in this study, narrow-sense heritability of transpiration efficiency based on grain yield was also higher in WDS than WW in durum wheat (Solomon and Labuschagne, 2004).

### Morphological, physiological, and phenological traits

Chlorophyll content and leaf relative water content were mostly controlled by dominant gene action in WW but were mostly controlled by additive gene action in WDS. CMS was almost equally governed by both additive and dominant gene action. This reveals that CMS depended on multiple morphological and physiological factors and many genes with different actions are involved in determining CMS. In accordance with this study, CMS is one of the main drought tolerance indicators in rice and durum wheat (Tripathy et al., 2000; Bajji et al., 2002) which is a polygenic trait in nature (Tripathy et al., 2000).

Fruit yield genetic control was mostly governed by additive gene action in both WW and WDS in the glasshouse (Table 5). But, narrow-sense heritability and GCA/SCA ratio of fruit yield in the field suggest that fruit yield was mostly controlled by dominant gene action in both field experiments. Also, inheritance of fruit yield was affected by environmental conditions. Therefore, breeding to improve fruit yield in coriander to cultivate in WW and WDS conditions will be difficult without selection for surrogate traits due to the complex genetic control of fruit yield.

Gene action influencing flowering time (Table 5) was in many ways similar to percent assimilate partitioned to fruit (Table 5) in both water treatments. The similar pattern of gene action for both flowering time and percent assimilate partitioned to fruit under WDS would allow a breeder to improve simultaneously the flowering time and percent assimilate partitioned to fruit in coriander. Also, if there was a significant genetic correlation between these traits, selection for one trait would lead to improvement in the other trait. Therefore, this result suggests that improvement of assimilate partitioned to fruit and flowering time under drought stress could be simultaneously achieved in a coriander breeding program (Khodadadi et al., 2016b).

### Genetic correlation among traits

Results indicate that increasing assimilate partitioning into roots leads to low fruit yield in WDS in both glasshouse and field conditions. Therefore, despite similar gene action controlling root traits and percent assimilate partitioned to fruit under WDS (Table 5), the hypothesis that drought avoidance by modifying root traits to maximize water uptake is a predominant drought resistance mechanism in coriander was rejected. However, Lopes and Reynolds (2010) suggested that this depends on water availability at depth. Drought tolerant genotypes of wheat can use alternative strategies, depending on whether water is available at depth. When this is the case, assimilate partitioning into deeper roots resulted in higher yield. However, where deep water is not available, assimilate partitioning in to grain is favored. Ratnakumar and Vadez (2011) reported that root depth and length density did not discriminate tolerant *Arachis hypogaea* L. genotypes from sensitive genotypes and related poorly to net water extraction. In that case tolerance to drought was mostly explained by capacity to maintain a high harvest index (percent assimilate partitioned to grain) under drought in tolerant genotypes (Ratnakumar and Vadez, 2011).

A significant negative correlation between flowering time and percent assimilate partitioned to fruit indicates that there is a trade-off between drought escape and drought avoid through maintaining water (Franks, 2011) in coriander and genotypes with early flowering time were able to produce acceptable fruit yield. In addition, the nature of gene action controlling flowering time and percent assimilate partitioned to fruit was similar. Therefore, drought escape through early flowering and fruit filling is a major drought resistance mechanism in coriander and simultaneous improvement of drought escape and fruit yield can be achieved under WDS. In this regard, Kazan and Lyons (2016) concluded that stressful growing conditions can lead to epigenetically transmitted genetic changes. Plants can rapidly evolve to select for drought escape and can successfully complete reproductive maturation. Therefore, selection for lower values of root traits and early flowering is required along with selection for higher fruit yield in WDS.

The genetic correlation results indicate that to improve fruit yield under drought stress, genotypes with lower percent assimilate partitioned to shoot must be considered. Conversely, genotypes with higher values of percent assimilate partitioned to fruit, chlorophyll content, leaf relative water content, and CMS must be given high importance. This is in accordance with Blum (2009) who concluded that high transpiration efficiency is not appropriate unless it leads to high harvest index under drought stress. Lal et al. (2006) and Sheshshayee et al. (2006) observed positive correlation between chlorophyll content and water use efficiency and transpiration efficiency, respectively. Similar to this study, Rahman et al. (2008) reported that CMS had a positive but not significant correlation with seed cotton yield under water stress.

### General and specific combining ability

The consideration that drought escape is a major drought resistance mechanism in coriander suggests that the parental genotypes P_1_ (TN-59-230) and then P_2_ (TN-59-160) would be the best parents among the parental genotypes tested to be used as donor parents to develop coriander varieties with high fruit yield under WDS environments. The P_1_ (TN-59-230) had high drought escape genetic potential likely because it originated from a naturally stressful climate (Bushehr; Hot desert (BWh) in Koppen classification) and showed 41 and 32 days earlier flowering time than the highly drought susceptible P_6_ (TN-59-158) and relatively high yielding and tolerant P_3_ (TN-59-160) under WDS. P_1_ contributed hybrids exhibited early flowering time. Also, the GCA effect of P_1_ for percent assimilate partitioned to fruit was higher than the other parents and the SCA effects of P_1_ contributed hybrids were high under WDS. These observations confirm that the P_1_ genome contained effective genes involved in drought stress escape and can successfully transmit these genes to progeny.

The GCA effects of the parents involved in the superior specific cross-combinations identified for all the target traits studied suggest that the SCA status of the crosses was influence by of the GCA effects of the parents involved which is contrary with Lal et al. (2006) results. In the case of percent assimilate partitioned to fruit (H_1×4_), leaf relative water content (H_1×3_), chlorophyll content (H_2×6_), fruit yield in the glasshouse (H_1×5_), and fruit yield in the field (H_1×3_); crossing between two parents which had a significant negative GCA and the greatest significant positive GCA resulted in the greatest positive (or negative) SCA effect under WDS. Therefore, considering the genetics of productivity traits and their genetic relationship with drought escape, it appears that the best approach would be to start a breeding program with crosses between high × low GCA types. This type of cross for desirable traits in coriander under WDS would be expected to lead to an appropriate segregating population allowing subsequent screening for simultaneous improvement of fruit yield and drought resistance.

## Conclusion

Overall, our results indicate that coriander genotypes may rely on different strategies in response to water deficit stress. Therefore, to improve coriander yield, selection should be done in conditions that are representative of the target region for breeding. Genetic control of fruit yield was complex. This suggests that secondary or surrogate traits could be used to improve coriander fruit yield. Results also suggest that to improve fruit yield under WDS, it would be possible to select for low percent assimilate partitioned to root, percent assimilate partitioned to shoot, root number, root diameter, root dry mass, and root volume as well as early flowering time while conversely selecting for high percent assimilate partitioned to fruit, leaf relative water content and chlorophyll content. According to GCA and SCA values it is recommended to cross between two parents having high and low GCA to start a breeding program. In this regard, P_1_ (TN-59-230) and P_2_ (TN-59-160) genotypes are suitable as donor parents.

## Author contributions

HD, MK, and MJ designed experiments, MK performed development of hybrids. MK participated field and glasshouse experiments and data collection. HD and MK analyzed the data and wrote the paper. All authors read and approved the final manuscript.

## Funding

This research funded by Tarbiat Modares University (Grant Number: D82/3564).

### Conflict of interest statement

The authors declare that the research was conducted in the absence of any commercial or financial relationships that could be construed as a potential conflict of interest.

## Supplementary material

Supplementary Figure 1 **Daily vapor pressure deficit (VPD) and rainy days after the beginning of the experiment. (A)** VPD (kPa) in glasshouse experiment at morning (07:00 h), midday (14:00 h), and night (21:00 h). **(B)** VPD and precipitation (mm) in field experiment at morning (07:00 h), midday (14:00 h), and night (21:00 h), and rainy days.

Supplementary Figure 2 **Water used in glasshouse and field experiments. (A)** Transpired water (ml) by genotypes in glasshouse. **(B)** Irrigation times of genotypes in field experiment. WW: well-watered; WDS: water deficit stressed; P_1_–P_6_: six parental coriander genotypes; H_1×2_–H_5×6_: 15 half-diallel hybrids.

Supplementary Table 1 **Soil properties of different layers of the experimental field**.
