# Supplementary figures and images for: Quantitative Genetic Analysis Reveals Potential to Genetically Improve Fruit Yield and Drought Resistance Simultaneously in Coriander
Source: Front Plant Sci. 2017 Apr 20;8:568. doi: 10.3389/fpls.2017.00568 (PMC5397498; doi:10.3389/fpls.2017.00568)

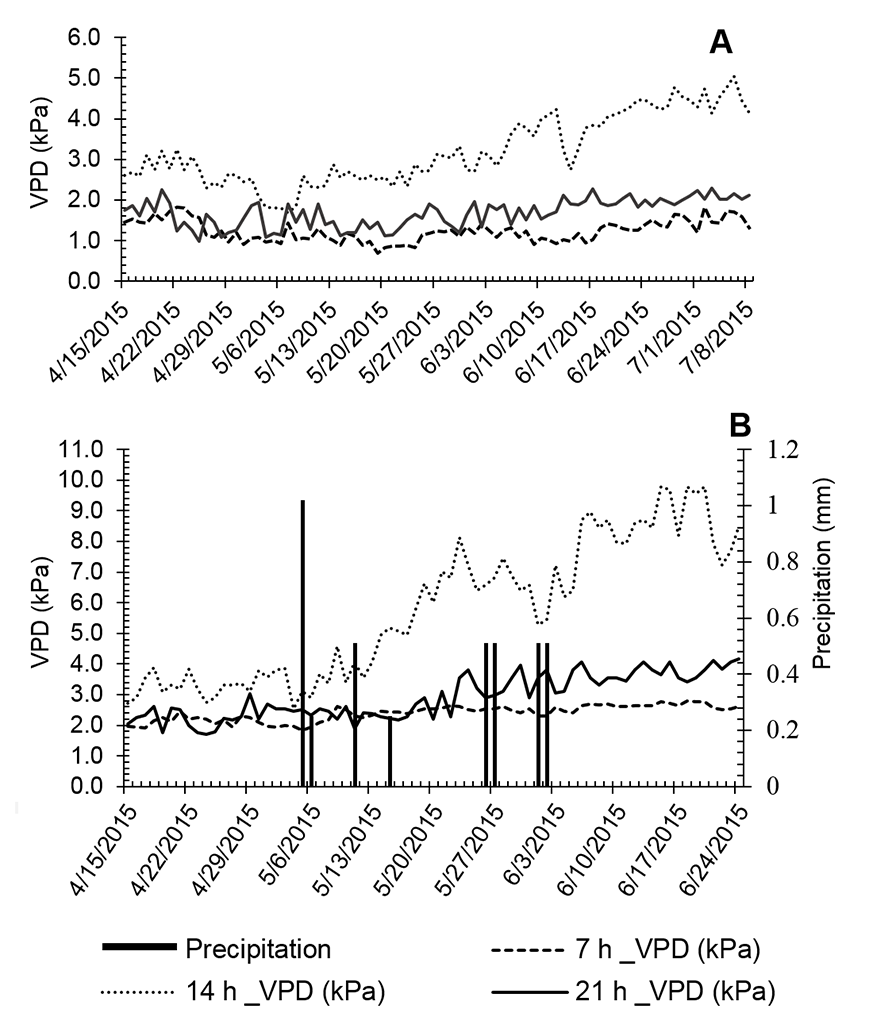

Supplement: Supplementary Figure 1 — Daily vapor pressure deficit (VPD) and rainy days after the beginning of the experiment. (A) VPD (kPa) in glasshouse experiment at morning (07:00 h), midday (14:00 h), and night (21:00 h). (B) VPD and precipitation (mm) in field experiment at morning (07:00 h), midday (14:00 h), and night (21:00 h), and rainy days. [file Image1.TIF]

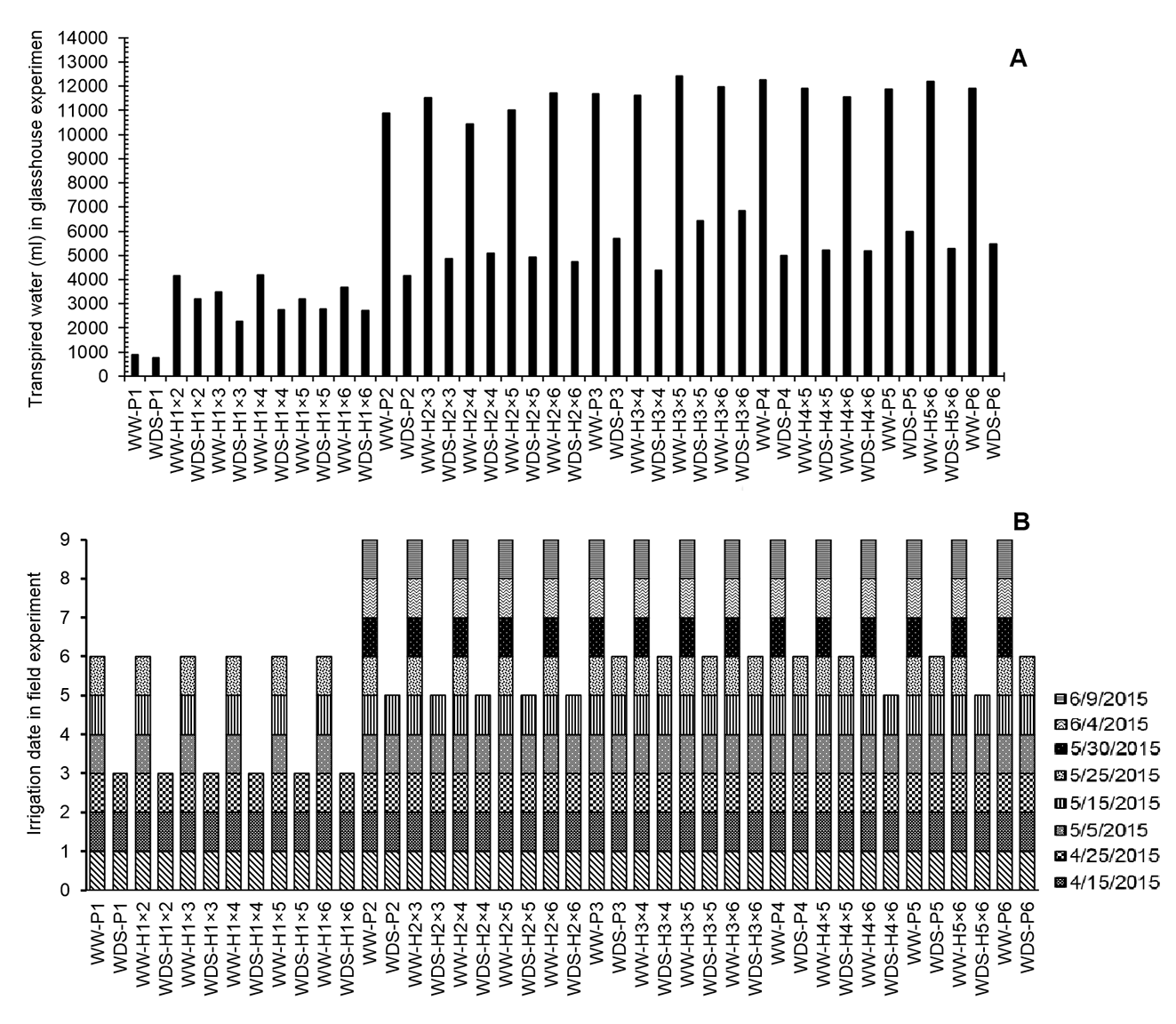

Supplement: Supplementary Figure 2 — Water used in glasshouse and field experiments. (A) Transpired water (ml) by genotypes in glasshouse. (B) Irrigation times of genotypes in field experiment. WW: well-watered; WDS: water deficit stressed; P1–P6: six parental coriander genotypes; H1×2–H5×6: 15 half-diallel hybrids. [file Image2.TIF]
