# Supplementary material for: Quantitative Genetic Analysis Reveals Potential to Genetically Improve Fruit Yield and Drought Resistance Simultaneously in Coriander
Source: Front Plant Sci. 2017 Apr 20;8:568. doi: 10.3389/fpls.2017.00568 (PMC5397498; doi:10.3389/fpls.2017.00568)
Supplement: Supplementary Table 1 — Soil properties of different layers of the experimental field. [file Table1.DOCX]

Supplementary Table S1. Soil properties of different layers of the experimental field

| Soil parameters | Soil depth (cm) | | |
| --- | --- | --- | --- |
|  | 0-20 | 20-40 | 40-60 |
| Sand (%) | 70 | 68 | 66 |
| Silt (%) | 15 | 18 | 18 |
| Clay (%) | 15 | 14 | 16 |
| Bulk density (g cm^−3^) | 1.2 | 1.4 | 1.48 |
| FC (%) | 16.5 | 19 | 15 |
| Organic C (%) | 1.61 | 1.45 | 1.09 |
| pH | 7.75 | 7.75 | 7.74 |
| EC (dS m^−1^) | 1.3 | 1.3 | 1.3 |
| Available N (kg ha^−1^) | 29.00 | 34.10 | 43.00 |
| Available P (kg ha^−1^) | 195.00 | 226.8 | 214.00 |
| Available K (kg ha^−1^) | 2085.0 | 2304.5 | 2465.9 |
